# All Silica Micro-Fluidic Flow Injection Sensor System for Colorimetric Chemical Sensing

**DOI:** 10.3390/s21124082

**Published:** 2021-06-14

**Authors:** Vedran Budinski, Denis Donlagic

**Affiliations:** Laboratory for Electro Optics and Sensor Systems, Faculty of Electrical Engineering and Computer Science, University of Maribor, Koroska Cesta 46, 2000 Maribor, Slovenia; denis.donlagic@um.si

**Keywords:** optical fiber sensor, spectral absorption microcell, colorimetry

## Abstract

This paper presents a miniature, all-silica, flow-injection sensor. The sensor consists of an optical fiber-coupled microcell for spectral absorption measurements and a microfluidic reagent injection system. The proposed sensor operates in back reflection mode and, with its compact dimensions, (no more than 200 µm in diameter) enables operation in small spaces and at very low flow rates of analyte and reagent, thus allowing for on-line or in-line colorimetric chemical sensing.

## 1. Introduction

Spectral absorption analysis provides a versatile way for the characterization of various fluids in a broad range of different fields such as physics, chemistry, biochemistry, and molecular biology. Spectrophotometry has thus carved out a distinctive position for itself in modern laboratories and industrial process control systems. Spectral absorption analysis possesses precision, sensitivity, accuracy, and selectivity.

Among spectral absorption sensing methods, principles utilizing colorimetric reagents provide one of the most versatile and simple ways to determine the various parameters of a liquid efficiently and quantitatively. There are a substantial number of different engineered color reagents that trigger color reactions in the presence of analytes, which are encountered in process control [1,2,3,4,5,6,7], environmental protection [8,9,10,11,12,13,14,15,16,17,18], biomedicine [19,20,21,22,23,24,25], the food industry [26,27,28,29], and other applications.

Development of optical sources, and especially optical spectrometers, reduced the cost and further enhanced out-of-the-laboratory use of these sensing systems. An optical absorption cell is an important element of any optical absorption measurement system. Optical fibers can provide very versatile ways to deliver light to and from the measurement location, have small dimensions, high chemical inertness and good compatibility with automatic/on-line measurement systems, and, thus, are very attractive for the development of robust sensors for a variety of chemical sensing applications [30,31,32,33,34,35].

A suitable and sufficient sample reagent delivery, absorption measurement and mixing system are needed to allow for efficient use of colorimetric reagents in on-line systems. These systems shall allow for system operation at very low consumption of color reagents. This requires a substantial miniaturization reagent delivery, mixing, and absorption measurement system, while allowing for full control and efficient mixing. Miniaturization is already employed in integrated microanalytical devices built on porous materials. These sensors, however, exhibit drawbacks, in the manner of a long response time, which can be up to a few hours [36], or limited or no reversibility, which leads to reagent replacement with suitable cleaning procedures that employ high temperature treatments [37].

Several options using optical fibers were investigated in the past to achieve the desired miniaturization. Currently, there are reported fiber optic sensors for colorimetric measurements of chemical parameters in water, such as sulfides, ammonia, nitrites, etc. However, the latter still either have bulky dimensions [18,38,39,40,41,42,43], which cause higher reagent and measured liquid consumption, or are built with nonstandard optical fibers and operate in in-line mode [44,45], which can present a limitation when operating in space limited areas.

Within this paper, we propose an in-flow colorimetric sensing system that combines an all-silica, tapered capillary reagent injector and an all-silica absorption microcell integrated with an optical fiber, which are inserted directly into the flow of the analyzed liquid. The system has the capability of spectral absorption measurements in both the visible and near-infrared (NIR) spectra, while relying on microfluidic color reagent injection. The proposed sensor facilitates online colorimetric liquid analysis with very low reagent consumption, thus producing small waste material quantities. The small dimensions of the proposed sensor enable installation of the sensor in a space limited environment, whereas the fiber technology facilitates remote operation and straightforward signal analysis by employing standard opto-electronic components. The proposed system might be especially interesting for all types of on-line water control management systems, for example, municipal, waste, surface, swimming, and industrial water preparation and control. Also, the all-silica sensor design provides good chemical compatibility with very different operating environments, provides the ability to operate at extreme temperatures (high or low), exhibits low susceptibility to temperature and is insensitive to electromagnetic interferences.

## 2. Sensor System Description

### Sensor System Design

**Figure 1:**
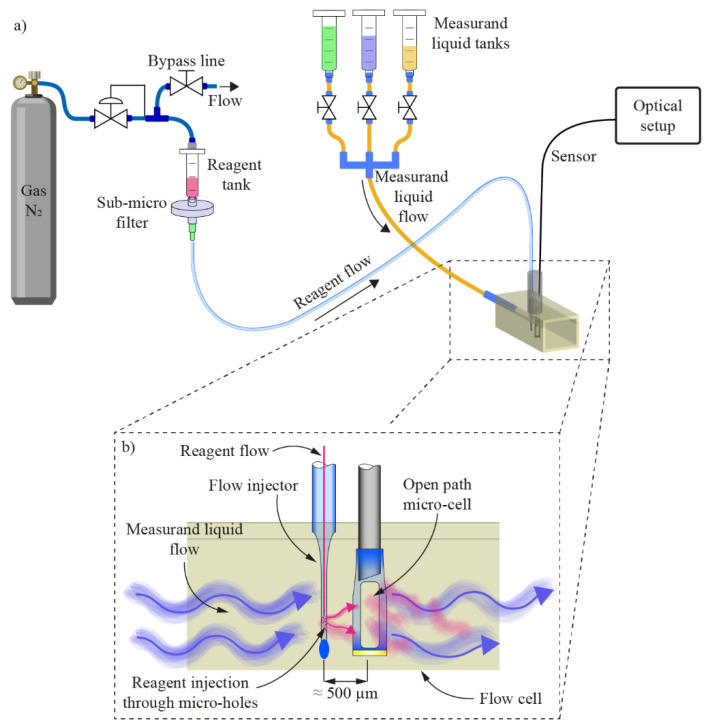
(**a**) Sensor system setup with spectral absorption subsystem and reagent flow injection subsystem. The spectral absorption subsystem is comprised of an open path micro-cell, a lead-in multimode optical fiber, a fiber coupler, a broadband fiber coupled light emitting diode and a spectrometer. The reagent flow injection subsystem consists of an all-silica flow injector connected to a pressurized reagent reservoir through a capillary with an inner diameter of about 85 μm. (**b**) The open path micro-cell and reagent flow injector are mounted into a custom fabricated test liquid flow chamber.

The proposed sensing system is presented in Figure 1a. It consists of spectral absorption and reagent flow injection subsystems. The spectral absorption measurement subsystem contains a miniature all-silica open-path microcell, a lead-in multimode optical fiber (105/125 μm, NA = 0.22), a fiber coupler, a broadband fiber coupled light emitting diode (BBLED) and a spectrometer. The microcell operates in a back reflection mode i.e., light emitted by the BBLED passes through the coupler and lead-in fiber to the microcell, where it back-reflects from the far end of the microcell towards the spectrometer. The reagent flow injection subsystem consists of a reagent reservoir that is pressurized at constant pressure by using a nitrogen cylinder and pressure regulator, an interconnecting capillary with an inner diameter of about 85 μm, and a microfluidic all-silica flow injector. N_2_ was used here only as a fluid drive system, and could be replaced by a micro-fluidic pump, compact air pump, or similar system. The flow injector and the microcell are placed in a cascade configuration, and in the direction of the analyzed liquid flow direction at the mutual distance of about 500 μm.

The outer diameter of the microcell is less than 200 µm, while the flow injector has a diameter of less than 40 µm. The micro cell is fabricated out of the lead-in fiber, a 3 mm long capillary (inner diameter of 128 µm and an outer diameter of 200 µm) and a flat-end cap consisting of a coreless fiber with a diameter of 200 µm. The cap is coated with a reflective layer to increase the magnitude of the back-reflected optical signal. For the liquid to enter the active area of the microcell, two side slits were cut along the active length of the glass capillary. The active area of the sensor is thus defined by the distance between the cleaved end of the lead-in fiber and the end-cap. The lead-in multi-mode fiber end-face is not perpendicular to the fiber axis, but is rather angled, to eliminate unwanted reflections that occur at the fiber-liquid interference when the sensor is submerged into the measured liquid, for example, in water. The optimum angle depends on the RI of the analyte. While the back refection can be reduced efficiently by substantial tilting of the fiber end-face, excessive tilt angles also reduce the amount of returned light signal, as the light propagation direction is refracted at the angled fiber-liquid interface, which can reduce the amount of light that can be returned by the cap/mirror surface located at the other end of the microcell strongly. The fiber end-face angle was thus determined experimentally by reading the back-reflected signal when changing the cleave angle gradually and submerging the fiber into the water. The angle that suppresses back-reflected light from the measured liquid/fiber interface to a negligible level, while still allowing for substantial back reflection from the microcell’s end-cap, proved to be about 9° to 10° in the case of a water-silica fiber system.

The flow injector has a diameter of 40 μm, and is about 2000 µm long, i.e., about the same size as the length of the slits on the microcell. The flow injector is permanently fused and closed on one side, and connected to a glass capillary with an inner diameter of 85 µm and outer diameter of 125 µm on the other. Along the flow injector, two micro holes were fabricated that served as reagent injection nozzles. The flow injector and the microcell were mounted into a custom fabricated aluminum holder to define the distance between both components to about 500 µm. The distance of 500 µm between the injector and the microcell ensured adequate mixing of a suitable volume of the analyzed fluid and reagent while providing the time needed for the reaction to occur. This distance was evaluated experimentally, and worked well for all used regents; however, reagents that react slowly with the analyzed fluid might require distances larger than 500 μm. The axes of both structures were placed in the same plane, while the nozzles were oriented at a 30° angle in respect to the flow direction, to facilitate better reagent and measured liquid mixing, as shown in Figure 1b. 

The pressurization of the lead-in flow injection capillary with the reagent at constant pressure provides constant and stable injection of reagent into the analyte flow, just in front of the microcell. The analyte with reagent is then carried by the flow into the microcell, where the spectral absorption is measured.

## 3. Test Setup

**Figure 2:**
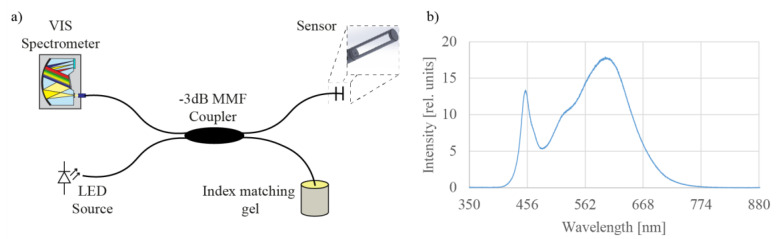
(**a**) Optical test setup comprising a miniature all-silica open-path microcell, a lead-in multimode optical fiber, a multimode fiber coupler, a broadband fiber coupled light emitting diode and a spectrometer. (**b**) Spectral characteristic of the light source.

The optical test setup is shown in Figure 2a. The light source was a low-cost high-brightness LED from Lumileds (type LXZ2-2280-3), with a broad emission spectrum, especially in the lower VIS wavelength range, i.e., between 420 nm and 470 nm (Figure 2b). Light from the light source was butt-coupled into the one 105/125 multimode fiber, which was further connected to a 3-dB coupler. For optical signal acquisition, a compact USB VIS spectrometer from Broadcom with a spectral resolution of 0.6 nm was utilized, which was connected to the second port of the coupler. A custom software for spectrum data analysis was developed in LabView.

**Figure 3:**
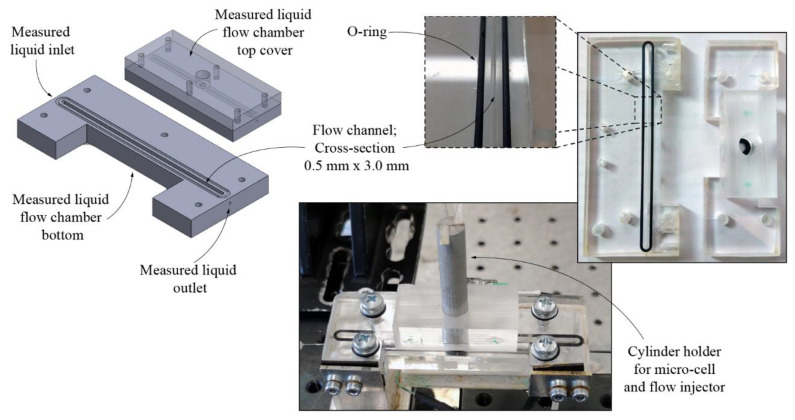
3D design and fabricated measured liquid flow chamber.

A dedicated test flow chamber with cross-section of 0.5 mm × 3.0 mm was fabricated out of PMMA and connected to the source of the test liquid. The sensor was placed into the flow channel in a way so as to be roughly in the center of the channel’s cross-section, as shown in Figure 3.

For observing the sensor and the reagent delivery system, a machine-vison camera with a long working distance microscope-lens system and background lighting was mounted on the sides of the flow chamber.

The setup for delivering the reagent into the reagent delivery system was comprised of a high-pressure cylinder with nitrogen gas, a mechanical pressure regulator, a bypass capillary (to allow for pressure regulation), a reference pressure transmitter, a syringe, and a high-grade sub micrometer filter (Figure 1). The syringe served as the tank for the reagent. The filter prevented particles with a diameter greater than 0.8 μm from entering into the capillary (tapered reagent delivery system), and, thus, potentially clogging it. Setting of the pressure at the pressure regulator, which was monitored by the pressure transmitter, ensured an adjustable, precise, and stable flow of the reagent.

A precise and constant analyzed liquid flow was achieved by siphoning. To allow for a controlled change in the composition of the analyzed liquid, three syringes were connected to the common inlet of the flow chamber. Flow from each syringe was controlled by adjusting the height of the latter and by un-pinching the outlet tubes. To prevent a pulsating flow due to the pressure changes induced by dripping out of the flow chamber outlet, an additional tube was mounted at the flow channel output and submerged into the waste liquid container.

## 4. Experimental Results

### 4.1. Micro Cell and Flow Injector Fabrication

**Figure 4:**
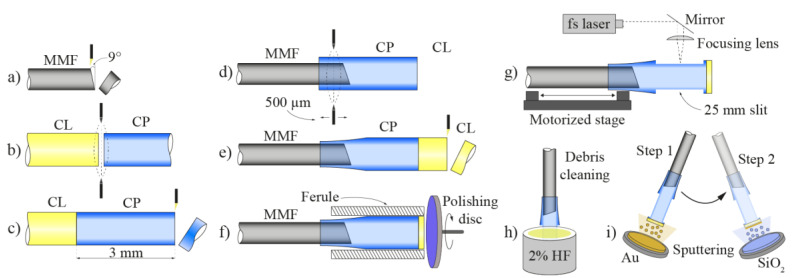
Microcell sensor fabrication stages, where the first steps employed preparation of the lead-in fiber (**a**) and the capillary with the end cap (**b**,**c**). Further steps utilized splicing the prepared capillary on the lead-in fiber (**d**), which followed adjusting of the thickness of the end-cap, first by cleaving (**e**) and afterwards by polishing (**f**) the coreless fiber end-cap. Furthermore, entry points (slits) for the analyte were fabricated by femtosecond laser micromachining (**g**), which was afterwards accompanied by cleaning of the leftover debris with a 2% solution of hydrochloric acid (**h**). The last step was sputtering the reflective layer on the outer end of the sensor’s end cap (**i**).

The complete microcell production procedure is depicted in Figure 4. The fabrication process consists of employing the cleaving, splicing, etching, micromachining, and sputtering techniques.

The process starts with cleaving the lead-in multimode fiber at an angle between 9° and 10° (Figure 4a). The next step includes preparing the capillary (CP) and the end cap. The capillary and the short section of a coreless fiber (CL) were cleaved and spliced together with a standard fusion splicer (Figure 4b). After completing the splicing process, the capillary was cleaved 3 mm away from the CL fiber (Figure 4c). Afterwards, the angle cleaved lead-in fiber was inserted into the cleaved capillary over the length of 500 µm (Figure 4d). With the use of a Vytran filament splicer, the capillary and MMF were heated and tacked together so that the capillary ‘shrank’ and ‘embraced’ the lead-in fiber uniformly along the length of about 500 µm. The latter assured a tight fit and good alignment between the lead-in MMF and the capillary. The next step included cleaving the coreless fiber end-cap (Figure 4e) and polishing it to the thickness of about 20 µm (Figure 4f). Entry points for the analyte were fabricated in the shape of two long slits with dimensions 2500 µm by 80 µm, which were positioned diametrically onto the capillary (Figure 4g). Slits were fabricated by employing a femtosecond laser micromachining system. Femtosecond lasers enable a variety of micromachining procedures, such as drilling, holing and hatching in transparent materials employing cold ablation [46] at the micro-scale. The technology facilitates fabrication of different optical fiber sensors [47,48,49]. After the micromachining process, the microcell was soaked for 40 s in a 2% solution of hydrochloric acid (HF), to clean the debris and any other silica leftovers in the active area of the microcell (Figure 4h). The last stage in the microcell fabrication process included the sputtering of a reflective layer on the outer end of the sensor’s end cap. Since the sensor is intended to operate in both acidic and alkaline environments, i.e., where the pH changes in the range of 2 to 14, gold (Au) was chosen as the reflective layer material [50]. The Au was also chosen as the reflective layer due to an acceptable reflectance [51]. For additional protection, a 0.6 μm thick layer of SiO_2_ was sputtered on the thin gold layer (Figure 4i).

**Figure 5:**
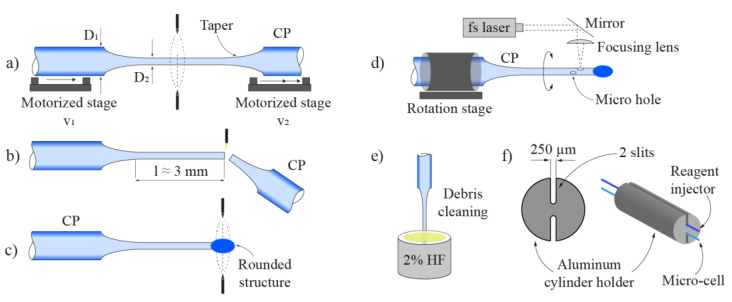
The reagent injector fabrication stages were comprised of tapering the glass capillary (**a**), which followed cleaving the tapered part (**b**) and fabricating a rounded structure at the tip of the cleaved capillary (**c**). Furthermore, two reagent micro-holes were fabricated by femtosecond micromachining in the tapered portion of the capillary (**d**). The final step was cleaning of the debris out of the holes with a 2% solution of hydrochloric acid (**e**). Microcell sensor and the reagent flow injector were inserted into a custom fabricated aluminum cylinder holder with two slits (**f**).

The injector fabrication process is presented in Figure 5. The first step in the fabrication process includes tapering the glass capillary (Figure 5a) from the initial 125 µm to about 40 µm outer diameter. Taper fabrication was conducted on a Vytran GPX-3000 Glass Fiber Processor. The next step was cleaving the capillary at the length of about 3 mm from the tapering zone, and fabricating a rounded structure at the tip of the cleaved capillary to achieve the hermetic sealing of the capillary end (Figure 5b). Fabrication of the rounded structure was conducted by employing a standard arc fusion splicer (Figure 5c), which was used to heat the cleaved tapered capillary far-end locally to form a ball end-cap. Afterwards, two micro holes with diameters of 30 µm were “drilled” with the femtosecond laser micromachining system. The micro-holes are located slightly beneath the sealed tapered capillary tip at a 30-degree angle from each other (Figure 5d). To prevent clogging of the micro holes with the leftover debris and silica particles, which arise from the micromachining process, the last step included the rinsing of the capillary with a 2% solution of HF (Figure 5e).

**Figure 6:**
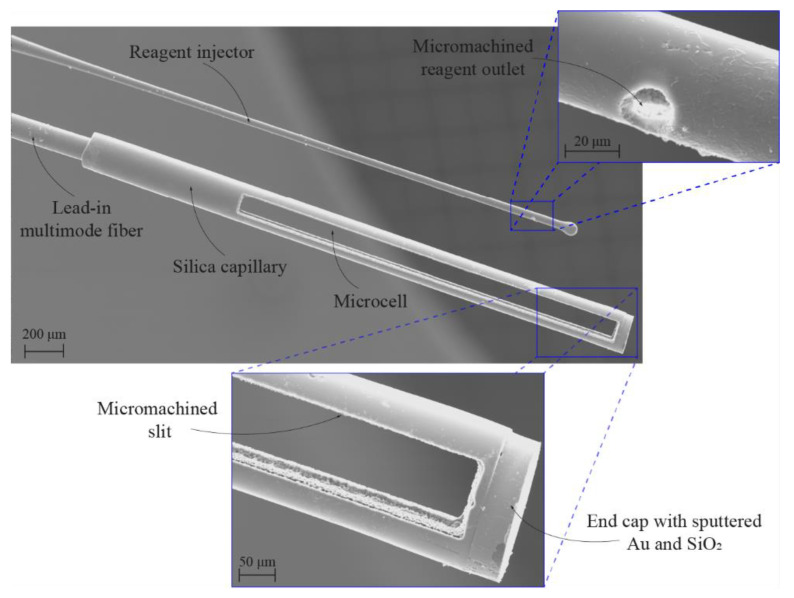
SEM image of the fabricated microcell sensor, which consists of a lead-in multimode fiber and a spliced-on capillary with micromachined slits for the analyte entry to the active area. Also shown is the SEM image of the reagent injector, which is fabricated from a tapered glass capillary with micromachined holes for the reagent outlet.

An aluminum cylinder holder was fabricated to form a complete flow injection sensor. The latter had two slits with widths of approximately 250 μm (for fitting the lead-in fiber and capillary with the buffer), which were fabricated with a wire erosion machine (EDM), thus allowing precise alignment and fitting of the sensor’s micro-cell cavity with the micro holes from the flow injector (Figure 5f). A Scanning Electron Microscope (SEM) image of the fabricated micro-cell and flow injector, as positioned by the aluminum holder, is shown in Figure 6.

### 4.2. Measured Liquid and Reagent Utilization

All measured liquids with their respective concentrations were prepared prior to the demonstration of the sensor operation and dispensed into the set of syringes. Switching between measured liquids with different analyte concentration was accomplished by interchanging flows from different syringes through the outlet pinch valves (Figure 1). To minimize the probability of cross contamination of the liquid with possible remaining residue from the preceding syringe, the analyte concentration measurement from the liquid dispensed in the subsequent syringe was instigated 60 s after engaging the flow. The choice of detecting pH, free chlorine, iron (Fe) or zinc (Zn) commenced after consulting the drinking water standards by the World Health Organization (WHO) and European Union (Council Directive 98/83/EC and the updated directive 2020/2184). A list of employed chemicals is provided in Table A1 (Appendix A) at the end of this paper. 

All experiments were accomplished at constant flow rates. Suitable flow rates were determined empirically by initial tests for each analyte. By adjusting the pressure of the nitrogen gas, the reagent flow range was between about 7.5 μL/min and 17 μL/min. The measured liquid flow was set by siphoning in the range between about 50 μL/min and 90 μL/min, thus reaching a fluid velocity of between 0.6 mm/s and 1.0 mm/s in the middle of the flow channel. The average response time of the sensor was below 15 s.

### 4.3. Optical Spectrum Evaluation and Initial Sensor Calibration

An evaluation of the measurement system was carried out by establishing a flow of measured liquids through the flow channel, while engaging in-flow reagent injection and recording of the back-reflected optical spectra of the sensor. Optical absorbance at the reagent-specific wavelength was then used as a parameter, which was correlated to a target analyte concentration.

The sensor was initially calibrated by recording the back-reflected light spectral characteristic when purified water was streaming through the flow chamber, without engaging the reagent flow. This spectral characteristic was stored in the program memory, and employed as the reference optical spectrum. After the initial calibration, the sensor does not have to be rinsed. Streaming the measured liquid through the flow chamber with the engaged reagent flow instigated a colorimetric chemical reaction, which affected the absorbance of light and, consequently, changed the back-reflected measured liquid optical spectrum. The absorbance was thus calculated by dividing the recorded measured liquid optical spectrum data with the reference optical spectrum data.

### 4.4. Demonstration of the Proposed Sensor Operation 

**Figure 7:**
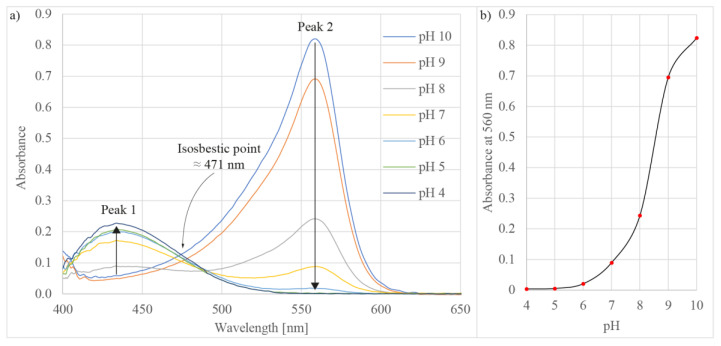
(**a**) Measured absorbance spectra of phenol red at different pH values and (**b**) absorbance versus pH at wavelength 560 nm.

In the first test, the proposed sensor was applied to pH measurements. To measure the pH, phenol red was used as the reagent. The latter was prepared at a concentration of 1 mg/mL, to achieve good solubility of the phenol red in purified water [52]. As the measured liquid, buffer solutions with values 4.0, 5.0, 6.0, 7.0, 8.0, 9.0, and 10.0 were employed in the test. Figure 7a depicts the typical measured absorbance spectra of phenol red at different pH values [53], where two significant peaks appear at wavelengths 430 nm (peak 1) and 560 nm (peak 2). Between the peaks is an isosbestic point at a wavelength of about 470 nm. When the pH changes, the amplitudes of the peak change reciprocally, i.e., when moving towards lower pH, the amplitude of peak 1 increases, whereas the amplitude of peak 2 decreases. When changing the pH of the solution, the absorbance at wavelength 560 nm (peak 2) exhibited a sharper response in comparison to peak 1 (wavelength 430 nm), therefore, peak 2 absorbance values served for the pH level evaluation/determination. Figure 7b shows the absorbance changes at different pH values when observing peak 2. The absorbance characteristic of phenol red at wavelength 560 nm exhibits a flat bottom and flat top characteristic [54]. Depending on the pH range, the sensitivities were, thus, different, i.e., in the pH range between 6 to 9, the sensor exhibited a sensitivity of about 0.1 pH change, whereas in the ranges between 4 to 6 and 9 to 10, the sensitivity was about 0.4 and 0.2 pH change, respectively. A demonstration of sensor operation, when altering solutions with different pH values (pH 7 to pH 5 to pH 10) is presented in video S1, provided as Supplementary Materials of this research.

**Figure 8:**
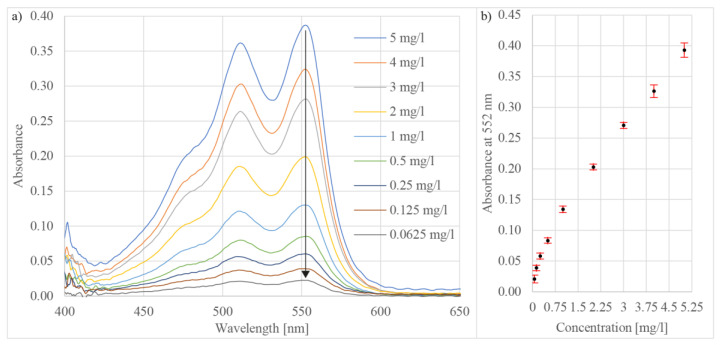
(**a**) Measured absorbance spectra of DPD at different free chlorine concentrations and (**b**) absorbance versus free chlorine concentration at wavelength 522 nm.

The next test demonstrated free chlorine concentration detection by application of the prosed system. The test employed N,N-diethyl-p-phenylenediamine (DPD) as the reagent. The reagent was prepared following the steps described in detail in [55]. Measured solutions with free chlorine at concentrations of 5 mg/L, 4 mg/L, 3 mg/L, 2 mg/L, 1 mg/L, 0.5 mg/L, 0.25 mg/L, 0.125 mg/L, and 0.0625 mg/L were prepared by adding the dichloroisocyanuric acid sodium salt dihydrate to purified water. The DPD reaction with chlorine results in an oxidation product semi-quinoid cationic compound, also known as Würster dye [56]. The Würster dye reaction generated two peaks in the absorption spectrum at wavelengths 511 nm and 552 nm (Figure 8a). The modulation height of the calculated absorption spectrum peaks decreased by reducing the free chlorine concentration. The absorbance was assessed from observing the peak at wavelength 552 nm (Figure 8b). The achievable detection limit of free chlorine was 62.5 μg/L, with a repeatability of below 1.2% RSD (n = 10).

**Figure 9:**
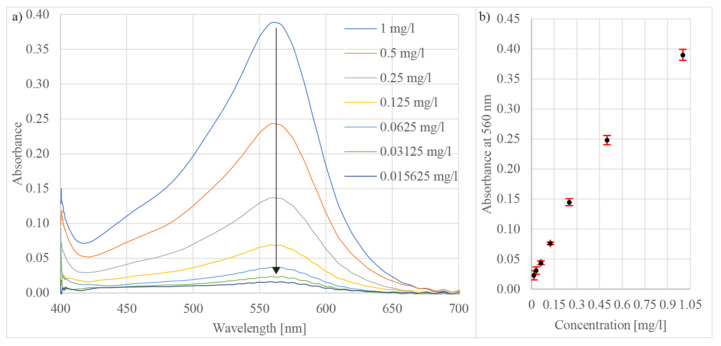
(**a**) Measured absorbance spectra of TPTZ at different iron concentrations and (**b**) absorbance versus iron concentration at wavelength 560 nm.

The sensor was also tested for concentration measurements of iron (Fe) in the water. The reagent for this test was based on tripyridyl-s-triazine (TPTZ), which was formulated as described in [57]. When the TPTZ reacted with iron, it generated a peak in the absorbance spectrum at about 560 nm (Figure 9a). To test the sensor’s sensitivity, measured solutions were mixed from purified water and iron(III) chloride (FeCl_3_) in concentrations from 1 mg/L to about 0.0156 mg/L (Figure 9b). The test indicated that the sensitivity of the proposed sensor facilitates detecting iron in concentrations below 0.02 mg/L with a repeatability of 1.6% RSD (n = 10).

**Figure 10:**
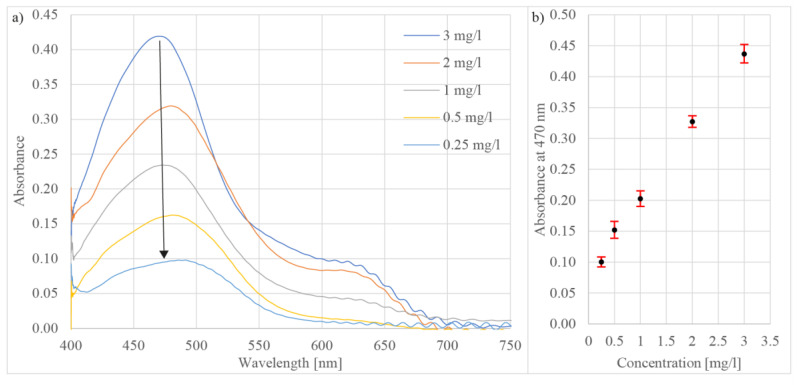
(**a**) Measured absorbance spectra of Zincon at different zinc concentrations and (**b**) absorbance versus zinc concentration at wavelength 470 nm.

In the last experiment, the sensor was tested for zinc (Zn) concentration determination in a water solution, with Zincon as the reagent, which was prepared as explained in [58]. To test the range and sensitivity, measured solutions based on purified water with added Zinc chloride (ZnCl_2_) were prepared at concentrations of 3 mg/L, 2 mg/L, 1 mg/L, 0.5 mg/L, and 0.25 mg/L. The chemical reaction between the reagent and zinc in the measured solution instigated two peaks in the calculated absorption spectrum, i.e., the first peak at 470 nm and the second one at 620 nm (Figure 10a). The modulation that appears in the red region of the spectrum arises from the refractive index change induced when the reagent and the measured liquid are mixed [59]. The modulation was pronounced differently when employing diverse reagents, and when the microcell was rinsed with clean water, the modulation disappeared. The concentration was calculated from the absorbance peak at wavelength 470 nm. The sensor exhibited sensitivity to zinc detection at 0.25 mg/L with repeatability less than 1.5% RSD (n = 10) (Figure 10b). The proposed sensor is capable of detecting Zinc in drinking water at the maximum levels (3 mg/L) specified by the WHO/EU Drinking Water Directive.

A result comparison of the proposed sensor with other reported colorimetric methods for detection of free chlorine, iron, and zinc is provided in Table 1.

## 5. Conclusions

This paper presented a miniature all-silica micro-fluidic flow injection sensor system for the reagent-based colorimetric analysis of liquids. To the best of our knowledge, the presented all-silica microcell and micro-injector concept for on-line colorimetric measurements is presented for the first time. The operation of the proposed system was demonstrated for measurement of the concentration of common analytes that are found in typical water-analysis related applications. The sensor exhibited high sensitivity, good stability, and measurement repeatability, overall, below 2% RSD. The flow rates of the reagent did not exceed 20 μL/min, whereas the flow of the measured liquid did not go above 100 μL/min, thus generating very small quantities of waste material, equivalent to less than 200 g of waste material per day under continuous operation. The latter could even be reduced further by optimizing (i.e., minimizing) the flow channel size, which was intentionally made larger than necessary to allow for visual observation of the flow process. Due to the low flows of the reagent and the measured liquid, the water pressure can be low, i.e., lower than in a typical water piping system, thus minimizing the possibility of damaging the sensor. By adding multiple reagent injectors, simultaneous detection of different analytes could be possible.

The optical sensor setup consists of a simple LED broadband light source and commercial low-cost VIS spectrometer, connected to a multimode fiber. Further sensitivity tuning and adaptation to the desired analyte measurement ranges could be achieved by adjusting the length of the micro-cell and flow rates. Multiparameter measurement (i.e., detection of more than one analyte) by a proper microfluidic switching of reagent flow is also a straightforward extension of the proposed system (in the current experiments, the same sensor and the same setup were used to test the response to all four demonstrated analyses; only reagents and test solutions were replaced manually).

Miniature dimensions, simple signal processing, the possibility to operate in on-line and in-line mode, with low reagent consumption, presents this sensor as a viable solution for chemical colorimetric sensing in the fields of drinking and surface water quality monitoring, industrial process control, aquaculture, agriculture monitoring, bio-medical analysis, and other aqueous media monitoring. The proposed system might also be applicable to gas phase sensing applications. This might, however, require adjustment of the microcell length and injection system, while providing suitable gas phase reagents.

## Figures and Tables

**Table 1:** Result comparison of the proposed sensor with other reported colorimetric methods for detection of free chlorine, iron, and zinc.

Parameter	Range (mg/L)	LOD (mg/L)	Reference
Free Chlorine	0.04–6.07	0.015	[60]
Free Chlorine	0.005–0.4	0.0015	[61]
Free Chlorine	0.07–5.0	0.0625	This work
Fe	1.4–3.4	0.028	[62]
Fe	0.0–0.616	0.0135	[63]
Fe	0.016–1.0	0.0156	This work
Zn	0.000653–6.5	0.000229	[64]
Zn	0.0–1.0	0.16	[65]
Zn	0.25–3.0	0.25	This work

## Data Availability

Not applicable.

## Supplementary Materials

The following is available online at https://www.mdpi.com/article/10.3390/s21124082/s1: Video S1: Demonstration of pH measurement: pH 7 to pH 5 to pH 10.

## Appendix A

**Table A1:** Chemicals employed in this investigation.

Chemical Name	Chemical Formula	CAS No.	Supplier
Iron(III) chloride	FeCl_3_	7705-08-0	Sigma-Aldrich
Zinc chloride	ZnCl_2_	7646-85-7	Sigma-Aldrich
Dichloroisocyanuric acid sodium salt dihydrate	C_3_Cl_2_N_2_NaO_3_ × 2 H_2_O	51580-86-0	Merck
Phenol Red	C_19_H_14_O_5_S	143-74-8	Sigma-Aldrich
Buffer solution pH 10.0			Fluka
Buffer solution pH 9.0			Fluka
Buffer solution pH 8.0			Fluka
Buffer solution pH 7.0			Merck
Buffer solution pH 6.0			Merck
Buffer solution pH 5.0			Merck
Buffer solution pH 4.0			Fluka
N,N-Diethyl-p-phenylenediamine sulfate salt	(C_2_H_5_)2NC_6_H_4_NH_2_ · H_2_SO_4_	6283-63-2	Sigma-Aldrich
Sulfuric acid solution	H_2_SO_4_	7664-93-9	Sigma-Aldrich
Triton X-114	(C_2_H_4_O)n C_14_H_22_O	9036-19-5	Sigma-Aldrich
Zincon	C_20_H_15_N_4_NaO_6_S	62625-22-3	Sigma-Aldrich
2,4,6-Tris(2-pyridyl)-s-triazine	C_18_H_12_N_6_	3682-35-7	Merck
Sodium acetate	CH_3_COONa	127-09-3	Merck
